# Breast cancer cell adhesome and degradome interact to drive metastasis

**DOI:** 10.1038/npjbcancer.2015.17

**Published:** 2015-10-28

**Authors:** Asif Rizwan, Menglin Cheng, Zaver M Bhujwalla, Balaji Krishnamachary, Lu Jiang, Kristine Glunde

**Affiliations:** 1 The Johns Hopkins University In Vivo Cellular and Molecular Imaging Center, Division of Cancer Imaging Research, The Russell H. Morgan Department of Radiology and Radiological Science, The Johns Hopkins University School of Medicine, Baltimore, MD, USA; 2 The Johns Hopkins University School of Medicine, The Sidney Kimmel Comprehensive Cancer Center, Baltimore, MD, USA

## Abstract

**Background:**

Although primary breast tumors are detected early in most cases, it is inevitable that many patients remain at risk for future recurrence and death due to micrometastases. We investigated interactions between the degradome and the adhesome that drive metastasis, and have focused on matrix metalloproteases (MMPs) within the degradome and integrins and E-cadherin within the adhesome.

**Aims:**

The aim of this study is to identify interaction networks between adhesion molecules and degradative enzymes in breast cancer metastasis.

**Methods:**

We compared non-metastatic (BT-474, T47D, MCF7) and metastatic (MDA-MB-231, SUM149, SUM159) human breast cancer cell lines and xenografts, in which we measured growth rate, migration, invasion, colony formation, protein expression, and enzyme activity *in vitro* and *in vivo*.

**Results:**

The metastatic breast cancer lines and xenografts displayed higher expression and activity levels of MMPs, which was also confirmed by noninvasive imaging *in vivo*. These metastatic breast cancer models also displayed elevated heterophilic cell-extracellular matrix (ECM) and lower homophilic cell–cell adhesion compared with those of non-metastatic models. This was conferred by an increased expression of the heterophilic cell adhesion molecule integrin β1 (ITGB1) and a decreased expression of the homophilic cell adhesion molecule E-cadherin. Inhibition of MMPs in metastatic cells led to a reduced expression of ITGB1, and stimulation of ITGB1 resulted in higher MMP activities in metastatic cancer cells, demonstrating reciprocal dependencies between degradome and adhesome. Re-expression of E-cadherin (CDH1) led to an increased expression of the precursor form of ITGB1.

**Conclusions:**

Our results point toward a concerted interdependence of MMPs, ITGB1, and CDH1 that is critical for breast cancer metastasis.

## Introduction

The occurrence of metastasis is the most life-threatening aspect of breast cancer. In the first 10 years after diagnosis, up to 12% of Stage I/II breast cancer patients, whose cancers are locally contained within the breast, die. However, 60% of Stage III patients, whose cancers have invaded nearby lymph nodes, and over 90% of Stage IV patients, whose cancers have spread to distant organs, die within 10 years after diagnosis.^1^ Cancer metastasis is driven by networks of proteolytic enzymes, collectively referred to as the degradome, and networks of adhesion molecules, collectively referred to as the adhesome.^2^ Matrix metalloproteases (MMPs) are key players of the tumor degradome.^3^ MMPs act as molecular scissors for cancer cells to chop and dice components of the extracellular matrix (ECM), such as collagen1 (COL1) fibers, leading eventually to a remodeled ECM that provides an escape path for cancer cells to metastasize.^3^ More than 23 MMPs have been identified in humans, including 6 membrane-type MMPs.^4^

Cancer cell to ECM and cell-to-cell adhesion is, to a large extent, conferred by integrins and E-cadherin.^2^ Integrin β1 facilitates cell–ECM adhesion and movement of tumor cells into the stroma and thus assists in local invasion within the primary site and growth in metastatic sites.^5^ At least 24 different integrin complexes are capable of binding to distinct ECM ligands.^6^ Integrin can also form signaling complexes with oncogenic Her2, Met, and EGF receptors, and it can recruit MMPs to the cell surface for local degradation of ECM in human breast cancer.^7^ E-cadherin promotes homotypic tumor cell–cell adhesion and provides intercellular contacts that confine tumor cells to the primary tumor site.^8^ MMPs, integrins, and E-cadherin are all implicated in breast cancer metastasis.^8,9^

Although previous studies have shown that integrin activation controls metastasis in human breast cancer^10^ and that the cancer degradome contributes to tumor progression, invasion, cell–ECM communication, and the metabolome,^11,12^ the interaction between these two groups of molecular players and its role in metastasis is just emerging.^13^ Our hypothesis that cell adhesion molecules interact with the degradome is supported by (i) co-localized expression of adhesion molecules such as integrins and E-cadherin and degradative enzymes such as MMPs,^14^ (ii) regulation of MMP activities by integrins and E-cadherin,^15^ (iii) regulation of the integrin-mediated adhesome by MMPs,^16^ (iv) binding of key integrins as well as MMPs to the same extracellular matrix (ECM) components,^17^ and (v) interaction between integrins, E-cadherin, and membrane-bound MMPs in cell communication in several types of cancer.^16,18^ Recent findings also revealed that several types of cancer cells have associations between adhesion and degradome molecules.^16^ For example, in melanoma cells, integrin α_V_β_3_ protein binds directly with MMP-2 and thereby localizes MMP-2 to cell surfaces, and hence assists in ECM, specifically collagen, degradation.^13^ In human melanoma cells, MMP-2 cleaves fibronectin into small fragments to enhance cell adhesion mediated by α_v_β_3_ integrin.^16^ The co-localization of MMP-14 (MT1-MMP) and integrin β1 is necessary for local invasion by human adenocarcinoma, epidermoid carcinoma, and fibrosarcoma cells.^14^ It was also shown that MMP-14 and integrin form protein complexes that regulate fibronectin remodeling in murine myoblast cells.^19^ In human osteosarcoma cell lines, integrin α_2_β_1_ positively regulates the expression of MMP-1 and collagen-1α1.^20^

Over the past 40 years, several generations of MMP inhibitors have been investigated to potentially treat cancer development and metastasis. The first-generation of MMP inhibitors such as marimastat, ilomastat (GM6001) and batimastat were hydroxamate-based inhibitors based on the structure of collagen.^21^ They inhibit several MMPs, such as MMP-1, MMP-2, MMP-7 and MMP-9, by directly binding to Zn^2+^ ions in the active site.^16^ Clinical studies with these hydroxamate-based broad-spectrum MMP inhibitors were ineffective at doses associated with musculoskeletal toxicity in a phase III trial for metastatic breast cancers that were stable after chemotherapy.^22^ The second generation of hydroxamate-based MMP inhibitors was more selective for specific groups of MMPs, such as for example MMI-166, which is a selective inhibitor of MMP-2, MMP-9, and MMP-14.^23^ However, they still suffered from the general limitation of hydroxamate-based MMP inhibitors that drug metabolism leads to the loss of the hydroxamate Zn^2+^-binding group and thus were ineffective in human trials.^22^ The second generation of non-hydroxamate MMP inhibitors such as rebimastat, tanomastat, and SB-3CT were developed to avoid metabolic inactivation.^24^ However, their efficacies were inconsistent and opposing outcomes were obtained depending on the timing of administration. The third generation of MMP inhibitors, which are specific to only one target and possess no zinc-binding group, is currently being evaluated preclinically.^25^ MMPs are also involved in cytokine and chemokine activation and inactivation, cell-surface-receptor cleavage and release.^26^ The full potential of MMP inhibitors can only be explored once all MMP functions and all MMP interactions with other important molecules in cancer such as growth factors, apoptotic mediators, and adhesion molecules are fully understood.

In this paper, we have performed comparative studies with a set of non-metastatic (BT-474, T-47D, MCF-7) versus metastatic (MDA-MB-231, SUM149, SUM159) human breast cancer cell lines and xenografts. Experiments were performed to determine growth rate, migration and invasion, colony formation, adhesion, aggregation, protein expression (western blot) and activity (zymography). Enzymatically activatable optical imaging probes were utilized to study *in vivo* tumor degradome activity and angiogenesis. The results provided novel insights into the molecular networks that comprise the cancer adhesome and degradome in breast cancers, and tested how their combined expression and activation drives cancer growth, invasion, ECM remodeling, and metastasis. Our results also suggest that, collectively, the adhesome and degradome molecules expressed in a given tumor can predict the metastatic risk of this primary tumor.

## Materials and methods

### Cell culture

Human breast cancer cell lines, MDA-MB-231, SUM149, SUM159, BT-474, T-47D, and MCF-7, were obtained from the American Type Culture Collection (ATCC, MD, USA). Cells were stably transfected with a construct containing cDNA of tdTomato as outlined in the Supplementary Materials and Methods under ‘Cell lines’.^27^ Human mammary fibroblasts were a kind gift from Dr Gary Luker at the University of Michigan. All cells were incubated at 37 °C with 5% CO_2_ in a humidified incubator.

Descriptions of cell-specific culture media are given in Supplementary Materials and Methods under ‘Cell type specific media used for breast cancer cell lines’. tdTomato protein expression was detected by fluorescence microscopy using a ×20 objective attached to a Nikon inverted microscope, equipped with a filter set for 528 to 553 nm excitation and 600 to 660 nm emission and a Nikon COOLPIX digital camera (Nikon Instruments, Inc, Melville, NY, USA).^27^

### MMP expression and relapse-free survival in breast cancer patient

The relationship of MMP gene expression and relapse-free survival was evaluated in an integrated multi-study breast cancer transcriptomic data set using Kmplotter (http://kmplot.com).^28^ Kaplan–Meier estimates of 10-year relapse-free survivals (RFS) were calculated with data collected from patients with systemic treatment. The median of gene expression was used to dichotomize data into high- and low-expression groups. Differences in survival curves were evaluated by log-rank test. Significantly different Kaplan–Meier estimators indicate longer RFS for patients with low expression of MMPs compared to the high-expressing group.

### Gene analysis of adhesome and degradome in breast cancer cell lines

A breast cancer microarray data set (GSE-16975) was analyzed where the breast cancer cell lines were grown to optimal cell densities for RNA extraction and hybridization on Affymetrix microarrays.^29^ The heat map was generated using the Gene-e matrix visualization and analysis platform (http://www.broadinstitute.org). The heat map represents changes in relative content of adhesome and degradome gene expression levels in 17 metastatic breast cancer cell lines (MDA-MB-231, SUM149, SUM159, MDA-MB-468, MDA-MB-436, MDA-MB-157, MDA-MB-175VII, MDA-MB-361, MDA-MB-435s, BT20, BT549, DU4475, HCC1937, Hs578T, SK-BR-7, SUM102, SUM1315MO2) and 11 non-metastatic breast cancer cell lines (BT-474, T-47D, MCF-7, BT-483, MDA-MB-415, MPE-600, SUM52PE, SUM44PE, ZR-75-1, MDA-MB-134VI, CAMA-1).

### Protein–protein interaction network

Network analysis of potential protein interactions among adhesome and degradome proteins, whose expressions were different in metastatic versus non-metastatic cell lines in the Gene-e analysis, was carried out using the STRING-9.1 (http://string-db.org) computational tool and database with a high confidence interval of 0.7.^30^ The STRING network, composed of the proteins of interest, is constructed based on genomic context, high-throughput experiments, co-expression, and scientific reports.^30^ The network nodes are proteins and edges represent the predicted functional associations. A red line indicates the presence of fusion evidence; a green line neighborhood evidence; a blue line co-ocurrence evidence; a purple line experimental evidence; a yellow line text-mining evidence; a light blue line database evidence; a black line co-expression evidence.^30^ Clustering algorithms (*K*_means_=2) were used to extract relevant modules.^30^ Inter-cluster edges are represented by dashed-lines.^30^

### *In vitro* assays

We performed a number of assays to characterize the tdTomato-expressing breast cancer cell lines used in our studies. Cell proliferation was assessed with the WST-1 assay. A series of protease activity and adhesion assays were performed such as zymography to assess MMP activities, cell adhesion assays, hanging drop assays, cell aggregation assays, clonogenic assays, quantitative reverse transcription PCR (qRT-PCR), immunoblotting protein assays, cell migration, and invasion assays. E-cadherin transfection was done using E-cadherin-GFP, which was a kind gift from Jennifer Stow (Addgene plasmid # 28009). Experimental details are provided in Supplementary Materials and Methods under ‘*In vitro* assays’.

### *In vivo* and *ex vivo* fluorescence imaging

Optical imaging was carried out using the Xenogen IVIS 200 Spectrum system. Enzymatically activatable optical imaging probes MMPSense-680 (NEV10126), and AngioSense-750 (NEV10011EX) from PerkinElmer (Waltham, MA, USA) were injected into the tail veins of mice growing orthotopic tumor xenografts according to the manufacturer’s protocol. MMPSense is activated by MMP-2, -7, -9, -12, -13, and -14. AngioSense injection enables imaging of tumor blood vessels and was used for normalization of uneven delivery of the probes to the tumor. Each mouse was imaged at 24 h after the injection with the IVIS camera settings at 1 and 2 s exposure time, binning factor of 8, field of view of 18.8 cm, and f number of 2. Then, the animals were killed and tumor xenografts and lungs were excised for *ex vivo* fluorescence imaging. Four to six 2-mm thick fresh tissue sections were cut from the primary tumor using an adjustable tissue slicer (Braintree Scientific, Braintree, MA, USA). Tumor sections and whole lungs were imaged with IVIS camera settings at 1- and 2-s exposure time, binning factor of 8, field of view of 6.4 cm, and f number of 2. All experiments were carried out according to the approved guidelines of the Institutional Animal Care and Use Committees (IACUCs) of the Johns Hopkins University.

### *Ex vivo* preparation of tumor sections and histopathology

Following *ex vivo* imaging, tissues were fixed in 4% paraformaldehyde for 24 h and embedded in paraffin blocks. Serial sections of 5-μm thickness were cut from the formalin fixed, paraffin embedded tissue blocks and floated onto charged glass slides (Super-Frost Plus, Fisher Scientific, Pittsburgh, PA, USA).^31,32^ A hemotoxylin and eosin stained section was obtained from each tissue block. Unstained sectioned were further processed for immunohistochemical detection of MMP-1, MMP-9, and ITGB-1 as detailed in Supplementary Materials and Methods under ‘Immunohistochemistry’.

### Quantification and statistical analysis

Statistically significant differences between quantitative measurements were analyzed by unpaired Student’s *t*-test. *P*<0.05 was considered statistically significant. Box-and-Whisker plots for cell migration, invasion, and adhesion were generated by BoxPlotR (Montréal, Québec, Canada), where the center lines show the medians, box limits indicate the 25th and 75th percentiles as determined by R software (Vienna, Austria), whiskers extend 1.5 times the interquartile range from the 25th to 75th percentiles, and outliers are represented by dots.^33^ A one-way analysis of variance was calculated from the number of migrated or invaded cells in all experimental groups. *Post hoc* comparisons using the Fisher least significant difference were explored to compare the mean of one group with the mean of another group. *P*<0.05 was considered statistically significant. *In vivo* MMP activity was measured for each pixel by calculating the ratio of MMPsense divided by Angiosense optical signal intensity using in-house software written in Matlab (Natick, MA, USA). Box plots for MMP activity were also generated using Matlab.

## Results

### Adhesome and degradome molecules in metastatic versus non-metastatic breast cancers

**Figure 1:**
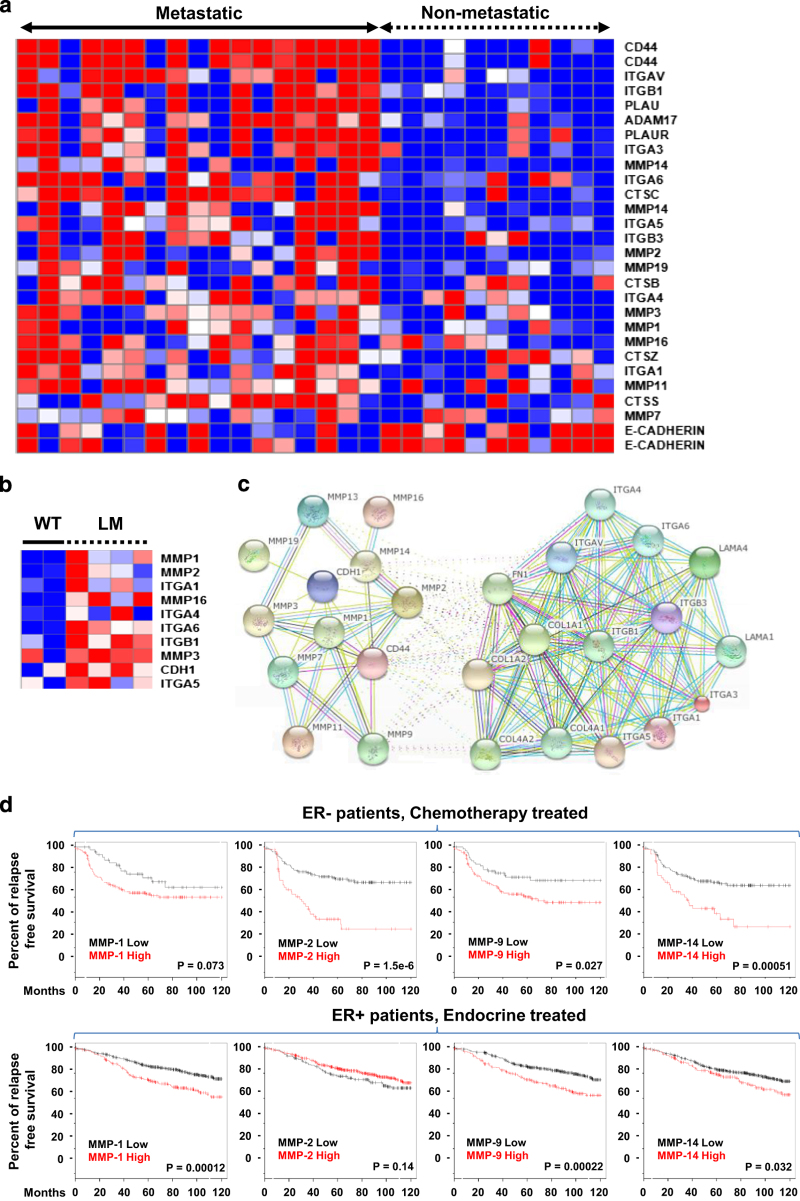
Analysis of publicly available microarray messenger RNA (mRNA) expression data from clinical breast tumors and breast cancer cell lines. (**a**) Expression levels of key adhesion molecules and key degradative enzymes discriminate between metastatic (*n*=17) and non-metastastic (*n*=11) human breast cancer cell lines (GSE16795). (**b**) MMPs, integrins and E-cadherin that are increased in subpopulations of lung-metastatic MDA-MB-231 (LM) compared with parental MDA-MB-231 (WT) cells (GSE2603). (**c**) Protein interaction network of major cell adhesion and degradome molecules differentially expressed in metastatic versus non-metastatic cell lines. (**d**) Kaplan–Meier curves show that high expression levels of MMP-2, MMP-9, and MMP-14 are predictive of decreased relapse-free survival in chemotherapy treated ER− human breast cancer patients, while high expression levels of MMP-1 and MMP-9 are predictive of decreased relapse-free survival in endocrine treated ER+ human breast cancer patients.

In an analysis of 1,144 genes from a panel of 28 human breast cancer cell lines given in the GEO data set GSE16795,^29^ we have demonstrated that metastatic compared with non-metastatic breast cancer cells contain significantly (*P*<0.01) increased messenger RNA (mRNA) gene expression levels of integrin α-1, α-4, α-5, α-6, α-V, and β-1, a decreased level of E-cadherin, and increased levels of MMP-2, -3, -11, -14, -16, and -19 as shown in Figure 1a. Some genes display significant changes in more than one microarray probe. To compare metastatic nodules with the corresponding primary breast cancers, we have analyzed the GEO data set GSE2603, where the lung-metastatic nodules of tail-vein-injected MDA-MB-231 breast tumor xenografts were expanded in cell culture. The lung-metastatic (LM) cell lines showed an increased gene expression of integrin α-1, α-4, α-5, α-6, α-V, and β-1, E-cadherin,and MMP-1, -2, -3, -11, -16, and -24 compared with wild-type MDA-MB-231 breast cancer cells (WT) as shown in Figure 1b. ^34^ It should be noted here that tail vein injected breast cancer cells represent a subpopulation of cancer cells with increased tendency to colonize a particular organ.^35^

### Analysis of interacting proteins within the degradome and adhesome

To identify known and potential protein–protein interactions relevant to the adhesome and degradome, we used the STRING 9.1 software and database. STRING quantitatively integrates protein interaction data from multiple sources for a large number of organisms. Using STRING, we identified several members of the MMP family (MMP-1, -2, -3, -7, -9, -11, -13, -14, -16, -19), integrin family (ITGA-1, -3, -4-, -5, -6, -V, ITGB-1, -3) and the E-Cadherin molecule that have direct and indirect associations with each other, as well as associations with major ECM components such as collagen, laminin, and fibronectin (Figure 1c). E-cadherin directly interacts with MMP-1, MMP-2, MMP-3, MMP-7, MMP-9, MMP-14, and MMP-19. ITGB-1 directly and indirectly interacts with MMP-2, -9, and -14. Overall, E-cadherin and the MMPs have a larger number of interactions and thus form a cluster. The integrins form a cluster with the major ECM molecules such as collagen, fibronectin, and laminin.

### Clinical relationship between the degradome and metastasis in breast cancer

Breast tumors with an overexpression of MMPs are associated with invasion and metastases.^9^ In our analysis of clinical data sets taken from the KM-Plotter database,^36^ we show that significantly increased expression levels of MMP-2 (*P*=1.5e−6), MMP-9 (*P*=0.027), and MMP-14 (*P*=0.00051) were predictive of a decreased RFS in chemotherapy treated ER- human breast cancer patients (*n*=211). High expression levels of MMP-1 (*P*=0.00012) and MMP-9 (*P*=0.00022) were predictive of a decreased RFS in endocrine treated ER+ human breast cancer patients (*n*=690) as shown in Figure 1d. Overall, our data analysis supports the roles of MMPs in breast cancer aggressiveness. High expression of MMP-2 (*P*=1.5e−6), MMP-9 (*P*=0.027), and MMP-14 (*P*=0.00051) is predictive of lower RFS in chemotherapy treated for ER− human breast cancer patients. High expression of MMP-1 (*P*=0.00012), and MMP-9 (*P*=0.00022) is predictive of lower RFS in endocrine treated ER+ human breast cancer patients. These Kaplan–Meier curves were generated using KM plotter from http://kmplot.com.

### Characterization of constitutively tdTomato-expressing metastatic and non-metastatic breast cancer models

We have performed comparative *in vitro* and *in vivo*/*ex vivo* experiments with human breast cancer cell lines and xenografts as outlined in Supplementary Figure 1A. All cell lines such as MDA-MB-231, SUM149, SUM159, BT-474, T-47D, MCF-7 were engineered to constitutively express tdTomato fluorescent protein for assessing tumor growth and metastatic spread by optical imaging *in vivo* and *ex vivo* (Supplementary Figures 1B–D). Proliferation assays of the tdTomato-expressing breast cancer cell lines demonstrated that all three metastatic lines grew significantly faster than the non-metastatic lines (Supplementary Figure 1E). Clonogenic assays of the tdTomato-expressing breast cancer cell lines were performed to examine whether a single cell can grow into a colony in uncoated, collagen1 coated, and matrigel-coated plates within 2 weeks (Supplementary Figures 2A and B^37^). Metastatic tdTomato-expressing MDA-MB-231 and SUM159 cells grew a significantly (*P*<0.01) higher number of colonies compared with all three tdTomato-expressing non-metastatic cell lines on uncoated, collagen1 coated, and matrigel-coated plates. Metastatic tdTomato-expressing SUM149 cells formed fa significantly (*P*<0.01) increased number of colonies on uncoated surfaces, but not in collagen1, and matrigel-coated surfaces, compared with all three non-metastatic cell lines.

**Figure 2:**
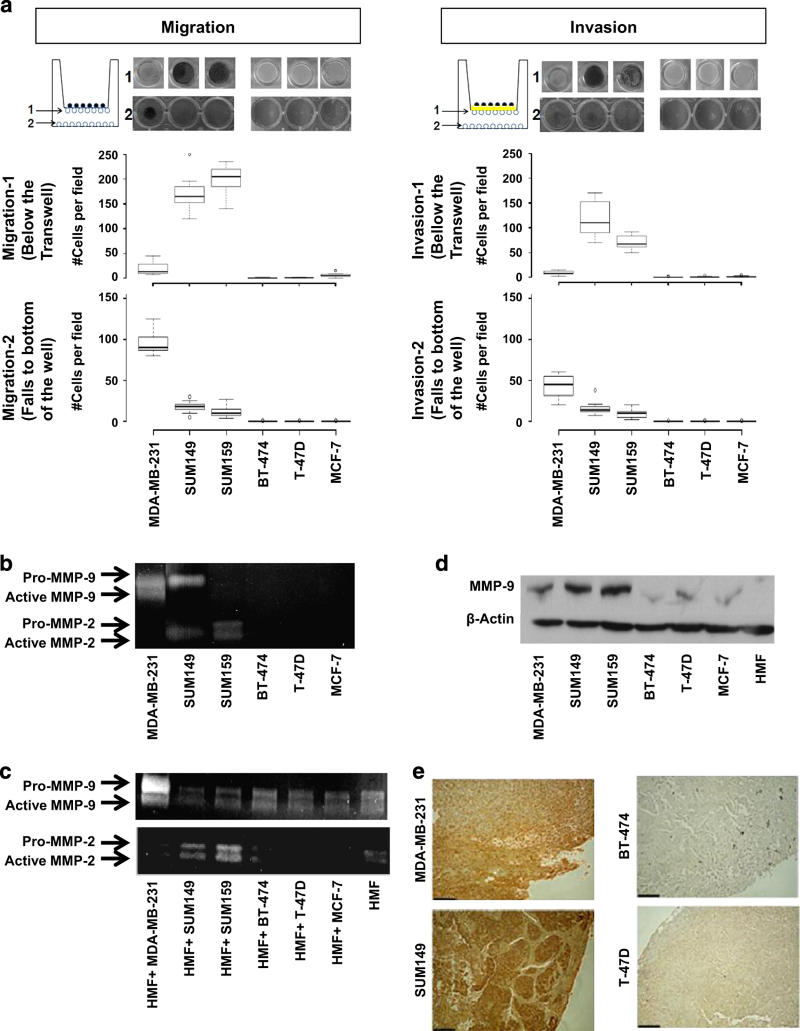
Invasion and MMP expression and activity in human breast cancer cell lines. (**a**) Breast cancer cell lines were allowed to migrate or invade matrigel for 48 h, and were stained with crystal violet. Representative photographs of the membrane insert and well from the transwell migration and invasion assays are shown. Average numbers of migrating and invading cells per field of view were plotted as Box-and-Whisker plots. Statistical testing for significant differences is shown in Supplementary Table 1. (**b**) *In vitro* MMP-2 and MMP-9 activity were measured using gelatin zymography. Conditioned media were collected after 24 h of incubation. Photographs show pro-MMP-2 (72 kDa), active MMP-2 (62 kDa), pro-MMP-9 (90–100 kDa), and active MMP-9 (82 kDa). (**c**) MMP-2 and MMP-9 activity measured by gelatin zymography of breast cancer cells that were co-cultured with HMF for 24 h. (**d**) Western blots showing MMP-9 expression in breast cancer cell lines. High MMP-9 expression was observed in metastatic lines, whereas non-metastatic lines and fibroblasts expressed small amounts or no MMP-9. ß-actin was used as loading control. (**e**) Immunohistochemistry analysis showing MMP-9 expression in sections from metastatic breast tumor xenografts. Scale bars, 100 μm.

We also tested the migration and invasion capabilities of all tdTomato-expressing cell lines. In transwell migration and invasion assays, significantly fewer non-metastatic tdTomato-expressing cell lines (BT-474, T-47D, MCF-7) migrated or invaded through the 8 μm pores of a transwell chamber compared with metastatic tdTomato-expressing cell lines (MDA-MB-231, SUM149, SUM159; Figures 2a and b). In these migration and invasion assays, around 90% of tdTomato-expressing SUM149 and SUM159 cells were detected on the bottom surface of the insert and the remaining 10% were attached to the plate well. For tdTomato-expressing MDA-MB-231 cells, around 20% of the cells migrated or invaded to the bottom surface of the insert, while the remaining 80% were attached to the well. All three metastatic cell lines showed higher migration and invasion compared with the migration and invasion by the non-metastatic cell lines (Supplementary Table 1). It should be noted here that the migration and invasion properties of tdTomato-expressing cells and wild-type cells were comparable for all of the six cell lines that we have used in our experiments (data not shown).

### MMP expression and activity profiles in metastatic versus non-metastatic breast cancer cell lines and xenografts

Breakdown of basement membrane is a critical step for tumor invasion. Loss of basement membrane type IV collagen is associated with increased activities of MMP-2 and MMP-9.^38^ To investigate the possible involvement of these proteases in our cell lines, gelatin zymography analyses from serum-free conditioned media were performed. As shown in Figure 2b, active MMP-9 was detected in medium conditioned by MDA-MB-231 cell and MMP-2 was detected in medium conditioned by SUM149 and SUM159 cells. Cancer-associated fibroblasts are also known to contribute to MMP activity *in vivo.* ^39^ We have examined the conditioned media of cancer cells co-cultured at a 1:1 ratio with human mammary fibroblasts (HMF) with gelatin zymography. HMF alone are able to produce pro-forms and active forms of MMP-2 and MMP-9. The activity of active MMP-2 was enhanced when HMF were co-cultured with MDA-MB-231, and active MMP-9 activity was enhanced when HMF were co-cultured with SUM149 or SUM159 cancer cells (Figure 2c). Western blot analysis of cell lysates revealed higher expression levels of MMP-9 in the metastatic cell lines, but no expression in the non-metastatic cell lines (Figure 2d). Immunohistochemical staining of tumor xenograft sections showed higher MMP-9 expression levels in the metastatic versus non-metastatic xenograft models (Figure 2e). Collagenase-type MMP-13 and membrane-type MMP-14 expression did not show any significant change across the tested panel of cell lines. However, collagenase MMP-1 and MMP-8 were significantly increased in metastatic cell lines as seen by immunohistochemistry (IHC) and western blot, respectively (Supplementary Figures 3A and B).

**Figure 3:**
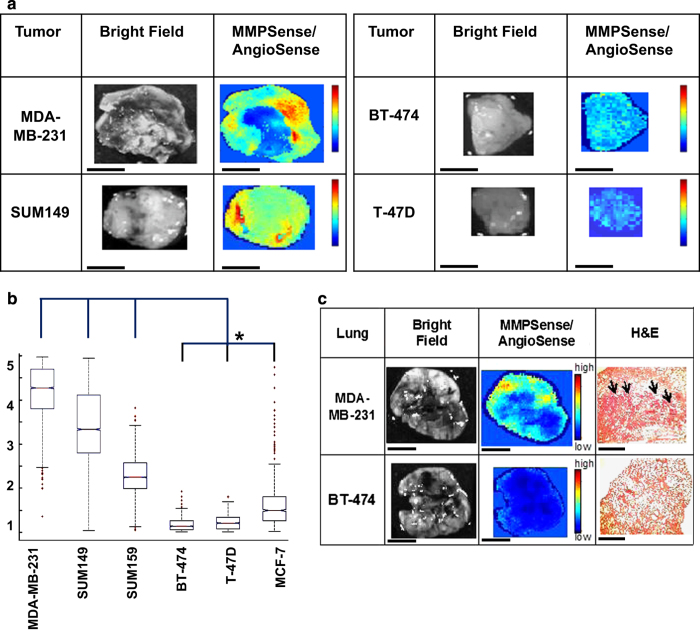
Analysis of MMP activities in breast cancer xenograft models. (**a**) *Ex vivo* fluorescence images of MMP activity in fresh breast tumor xenograft slices measured following MMPSense and AngioSense injection. Scale bar, 5 mm. (**b**) Box plots show the intensity of MMP fluorescent signals normalized to AngioSense to normalize for uneven delivery of the probe. *P<0.05. (**c**) Lungs of mice growing metastatic breast cancer xenografts displayed increased MMP activity. Scale bar, 5 mm. Corresponding H&E stains showed micrometastases in these lungs as pointed out by arrows. Scale bar, 2.5 mm.

Using MMP-activatable fluorescent imaging agents that are activated by key MMPs, we observed that metastatic breast tumors displayed increased MMP activities compared with non-metastatic breast tumors, which was shown in fresh 2-mm thick tumor sections *ex vivo* in Figure 3a. The resulting quantitative MMP activities, normalized to perfusion to account for agent delivery to the tumor, are shown in a box plot in Figure 3b. The lungs of mice growing primary tumor xenografts were also imaged for MMP activity. MMP activity in the lungs of mice with metastatic primary tumors was evident prior to metastatic seeding in the lungs, indicating that the secretion of degradative enzymes by the metastatic tumor or stroma occurs well in advance of metastatic seeding in distant organs, which is in good agreement with previous studies.^40^

### Strong heterophilic and weak hemophilic adhesion signature in primary metastatic breast cancer cell lines and tumor xenografts

**Figure 4:**
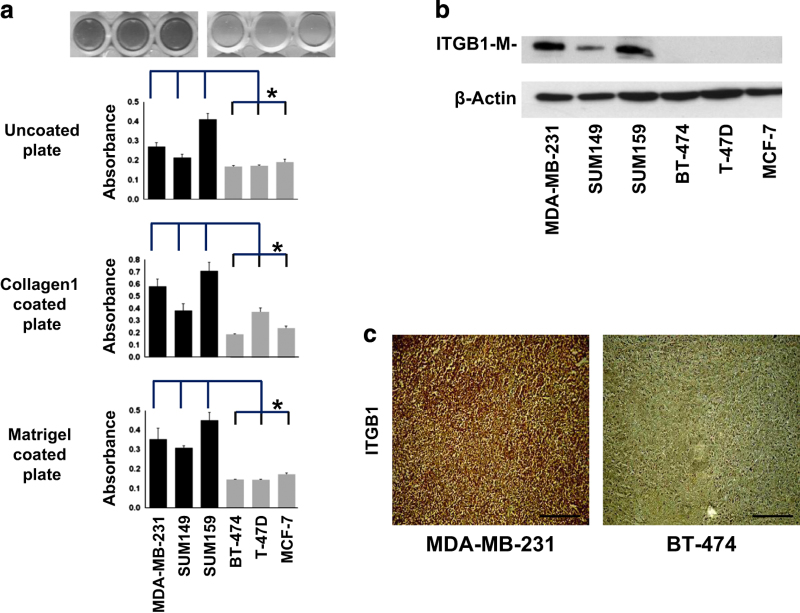
Analysis of cell adhesion and integrin β1 expression in breast cancer cell lines and xenograft models. (**a**) Representative photographs of cell adhesion assays where plates were coated with collagen1 (50 μg/ml). The column graphs represent quantitative cell adhesion assays on uncoated, collagen 1 coated, and matrigel-coated plates. Light absorbance from the WST-1 assay, which is proportional to the number of cells adhering to the plate, is displayed on the *y*-axis. Values are mean±s.d. *P<0.05. (**b**) Immunoblots showed significant differences in mature integrin β1 expression in highly expressing metastatic cells compared with non-expressing non-metastatic cells. (**c**) Immunohistochemistry analysis demonstrated strong integrin β1 expression in metastatic MDA-MB-231 tumor sections, but not in non-metastatic BT-474 tumor sections. Scale bar, 200 μm.

**Figure 5:**
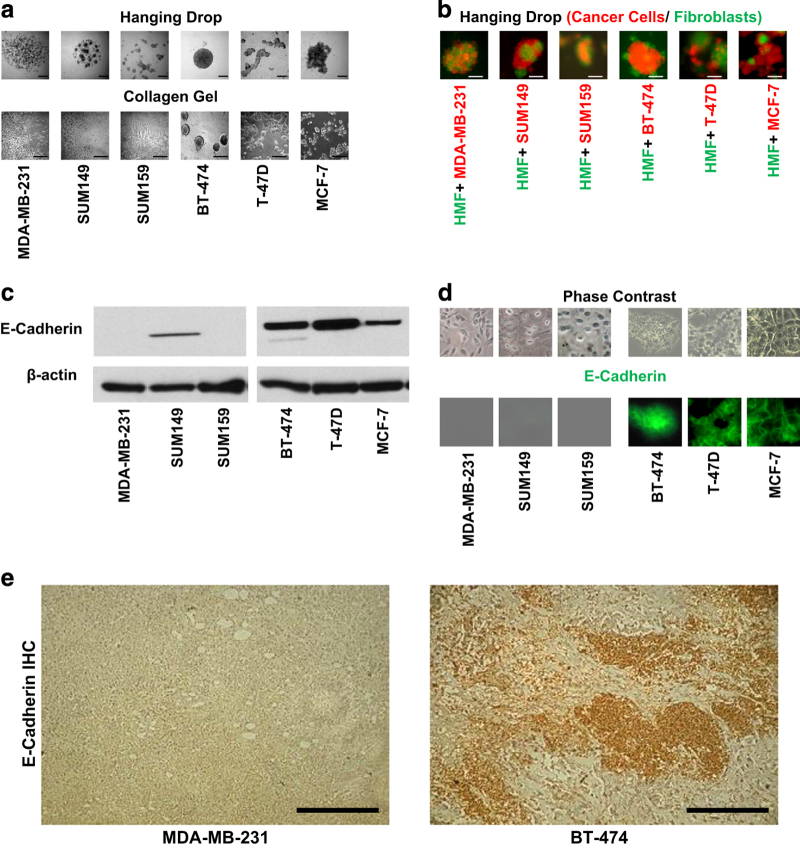
Homophilic and heterophilic cell adhesion characteristics and E-cadherin expression in breast cancers. (**a**) Metastatic cell lines showed lower cell aggregation in hanging drop assays and collagen 1 gel as compared to non-metastatic cell lines. Scale Bar, 200 μm. (**b**) Equal numbers of red-labeled breast cancer cells and green-labeled fibroblasts were mixed and evaluated in the hanging drop assay. Metastatic cells adhered to fibroblasts and formed a mixed population. Non-metastatic cells remained aggregated in a sphere and separated from fibroblast cells. Scale Bar, 200 μm. (**c**) Western blots showed significant differences in E-cadherin expression in lowly expressing metastatic cells compared with highly expressing non-metastatic cells. (**d**) Immunofluorescence staining demonstrated lower expression of E-cadherin in metastatic cells as compared to non-metastatic cells. (**e**) Immunohistochemistry analysis showing weak E-cadherin expression in metastatic MDA-MB-231 tumor sections, and high E-cadherin expression in non-metastatic BT-474 tumor sections. Scale bar, 200 μm.

Metastatic cells displayed increased adhesion compared to non-metastatic cells on ECM surfaces as measured by cell adhesion assay shown in Figure 4a. The immunoblots of cells, as well as immunohistochemistry (IHC) of tumor xenografts, consistently demonstrated an increased expression of the heterophilic adhesion molecule integrin β1 (ITGB1) in metastatic tumors compared to non-metastatic tumors (Figures 4b and c). Metastatic cell lines showed decreased cell aggregation in the hanging drop assay and in collagen1 gel (Figure 5a). When co-cultured with HMF, metastatic cells displayed increased adhesion to fibroblasts in the hanging drop assay (Figure 5b). Compared with non-metastatic cells, metastatic cells had a significantly decreased expression level of the homophilic adhesion molecule E-cadherin (CDH1) both *in vitro* and *in vivo *(Figures 5c–e).

### Interaction of degradome and adhesome

**Figure 6:**
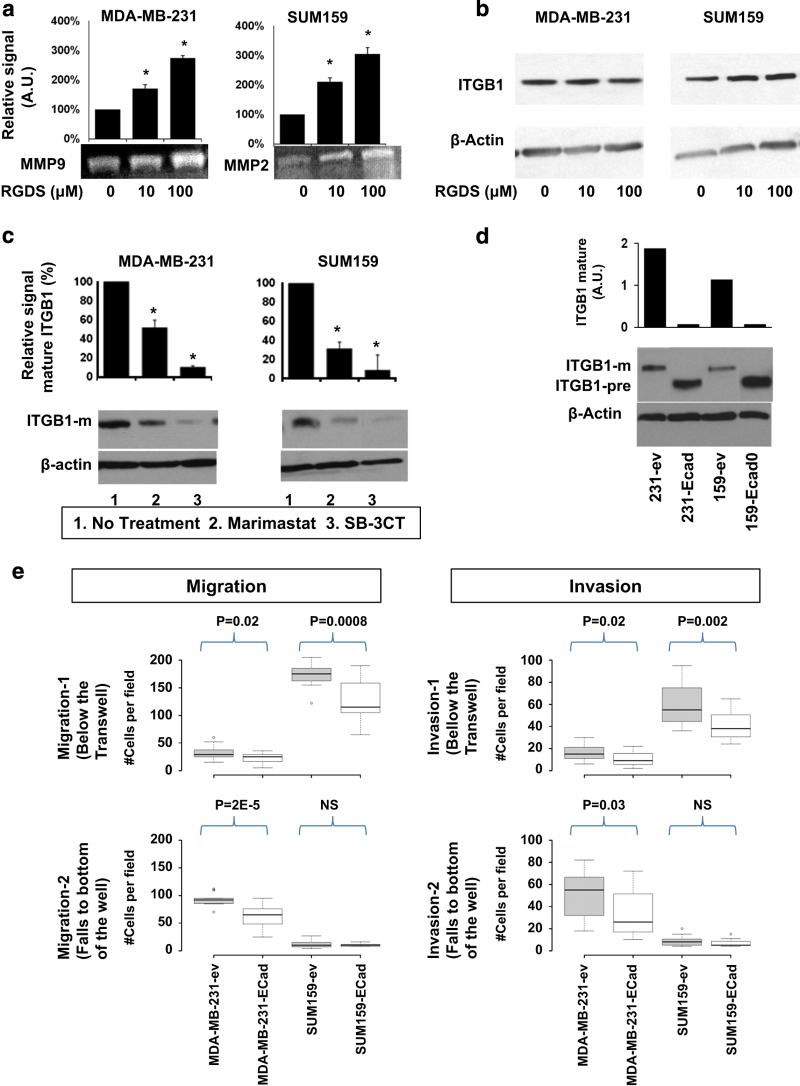
Interaction of MMPs, integrin β1, and E-cadherin in metastatic breast cancer cells. (**a**) MMP-9 and MMP-2 activities in conditioned media from metastatic breast cancer cell lines were analyzed at 48 h after treatment with the integrin β1 binding tetrapeptide RGDS. Secreted MMPs increased with increasing concentrations of RGDS. Values are mean±s.d. *P<0.05. (**b**) Cellular integrin β1 protein, as analyzed by western blot, did not show any differences when the cells were treated with RGDS. (**c**) Cellular integrin β1 protein levels significantly decreased when metastatic breast cancer cells were treated with the broad-spectrum MMP inhibitor Marimastat or with the MMP-2 and MMP-9 specific inhibitor SC-3BT for 48 h. Values are mean±s.d. *P<0.05. (**d**) E-cadherin re-expression in metastatic MDA-MB-231 and SUM159 breast cancer cells resulted in reduced expression of mature integrin β1 and elevated expression of the precursor form of integrin β1 as compared with empty vector (ev) transfected control cells. (**e**) E-cadherin re-expressing metastatic cell lines displayed reduced migration and invasion as compared with the corresponding empty vector (ev) control cells.

The major integrin β1 binding site is an Arg–Gly–Asp (RGD) peptide, which is present in a variety of ligands, which are part of the ECM such as collagen, laminin, and fibronectin and thereby represents a major recognition system for cell adhesion.^41^ We analyzed the effect of an RGD-containing peptide, namely Arg–Gly–Asp–Ser (RGDS), on the regulation of MMP secretion in cultured cells. When RGDS peptide was added to cell culture medium, the secreted gelatinases MMP-2 and MMP-9 increased in the metastatic MDA-MB-231 and SUM159 cell lines, as shown in Figure 6a. However, cellular integrin β1 protein, as analyzed by western blot, did not show any differences in expression level when the cells were treated with various concentrations of RGDS (Figure 6b). On the other hand, the mature form of integrin β1 protein significantly decreased (*P*<0.01) when metastatic MDA-MB-231 and SUM159 cells were treated with the broad-spectrum MMP inhibitor marimastat or with the MMP-2 and MMP-9 inhibitor SB-3CT for 48 h (Figure 6c). This suggests a reciprocal regulatory relationship between MMP-2/MMP-9 and integrin β1. It should be noted that the fourth amino-acid S in RGDS contributes toward the stability of the RGDS confirmation to fit the integrin receptors.^42^ Cells that were cultured in medium containing RGD sequence alone did not show any changes in the amount of MMP secretion.

Next, we transfected MDA-MB-231 and SUM159 cells to re-express the E-cadherin gene.^43^ Although we were able to detect E-cadherin gene expression by qRT-PCR in the transfected cells (Supplementary Figure 3), E-cadherin protein was not detected by western blot, most likely due to post-translational E-cadherin degrading mechanisms that are present in metastatic breast cancer cells.^44,45^ Nevertheless, we observed that E-cadherin re-expression at the mRNA level reduced protein expression of the mature form of integrin β1 in metastatic MDA-MB-231 and SUM159 cells (Figure 6d, Supplementary Figure 4). A significantly lower number of cells with E-cadherin re-expression were able to migrate or invade in transwell assays (Figure 6e).

## Discussion

Our study presents several important findings: (i) Increased expression and activity of MMP-2 and MMP-9 among others in metastatic breast cancer cell lines, xenografts, and lungs as compared with non-metastatic lines. (ii) Heterophilic adhesion, likely mediated by integrin β1, is increased in metastatic compared to non-metastatic lines. (iii) Homophilic adhesion, likely mediated by E-cadherin, is increased in non-metastatic compared to metastatic lines. (iv) Re-expression of E-cadherin reduced the expression of the mature form of integrin β1 in metastatic breast cancer cells. (v) A reciprocal interaction exists between integrin β1 and MMP-2/MMP-9 in metastatic breast cancer cells.

We observed that MMP-2 and MMP-9 activities, among other MMP activities, are increased in metastatic breast cancer cell lines, xenografts, and lungs as compared with non-metastatic lines. Our analysis of publicly available clinical and cellular expression data revealed that low levels of MMP-1, -2, -9, and -14 are important for breast cancer survival. However, a slew of other MMPs also confer breast cancer metastasis, depending on the particular cell lines studied. Since elevated MMPs are an important component of many aggressive tumors, it is a potential drug target for cancer therapy.^9^ Despite promising preclinical data, clinical trials using MMP inhibitors resulted in inconsistent outcomes. As evident from our data and the data of others,^40^ a key issue is that the types and levels of MMP expression and activity are quite variable across different breast tumors and the derived breast cancer cell lines, which is one of the main reason for the inconsistent outcomes in clinical trials using MMP inhibitors. However, systemic treatment with MMP inhibitors would be a good way of treating breast cancer metastasis, as MMP activity in metastatic sites is upregulated during or even prior to metastatic seeding,^40^ as also observed in the lungs of our metastatic tumor xenograft model.

We showed that mature active integrin β1 expression was elevated in metastatic breast cancer cell lines that adhered to the ECM components collagen 1 and matrigel, while non-metastatic cell lines contained no integrin β1 and did not adhere well. We also observed that metastatic breast cancer cell lines displayed increased heterophilic adhesion. It is known that integrin heterodimers containing β1 subunits are receptors for various types of ECM molecules such as collagens, laminins, fibronectin, and tenascin, and thus have an essential role in cell–ECM adhesion.^46^ In addition, β1 integrins initiate signaling cascades in the cell in response to extracellular chemokines (outside-in), and also transmit intracellular signals that change the way the cells interact with the ECM (inside-out).^6^ These signaling pathways regulate cell adhesiveness by changing the conformation of β1 integrin binding to the ECM.^5^ These dynamic adhesion processes are crucial for conferring the migration abilities of cancer cells, which degrade mammary basement membrane, the dense ECM surrounding the tumor, and the ECM of the walls of blood vessels.

In our study, E-cadherin was increased in non-metastatic breast cancer cell lines that adhere well to each other but not to fibroblasts, as compared with metastatic breast cancer cell lines. This finding is in good agreement with the suggestion that dynamic E-cadherin-mediated cell–cell adhesions and integrin-mediated cell–ECM adhesions govern the invasive and metastatic potential of tumors.^8^ Loss of E-cadherin results in the weakening of cell–cell adhesion.^8^ On the other hand, increase in integrin-β1 subunits mediates cell–ECM interactions by linking signals from the environment to the actin cytoskeleton.^6^ Simultaneously, these two processes enhance the agility of metastatic breast cancer cells, which let them respond to external signals and execute successful migration and invasion.^8^

We observed that E-cadherin gene re-expression in metastatic breast cancer cells reduced the amount of mature integrin β1 protein in metastatic breast cancer cells. In the clinical setting, it was shown that the secondary metastatic site can induce the re-expression of E-cadherin in cancer cells, which is a critical step in the survival of cancer cells in the new microenvironment.^47^ The effect of re-expressing E-cadherin on integrin β1-subunits has, to the best of our knowledge, not been studied so far. Our data suggest that reducing the amount of active integrin β1 increases the expression of E-cadherin. Taken together, our data indicate that a reciprocal interaction exists between E-cadherin and integrin β1, which couples homophilic cell–cell and heterophilic cell–ECM adhesion in metastatic breast cancer cells. This will enable metastatic breast cancer cells to lose contact to other cells at a time when they start binding to the ECM while they migrate, and switch back to binding to cells instead of ECM when they arrest and colonize distant sites.

We found that RGD-stimulation of metastatic breast cancer cells upregulated the expression of MMP-2 and MMP-9. *Vice versa*, when we inhibited MMP activities in cell culture by either Marimastat or SB-3CT, the expression of integrin β1 was significantly reduced. Our experiments with the MMP inhibitors Marimastat and SB-3CT showed for the first time that inhibition of MMP activities can reduce the expression of active integrin β1, suggesting an association between active MMP-2/MMP-9 and integrin β1 protein expression in metastatic breast cancer cells. It has previously been shown in human umbilical vein endothelial cells that the physical associations of MMP-2 with integrin β1 can promote ECM degradation by these endothelial cells.^48^ In good agreement with these findings, our protein–protein interaction network analysis of human data also predicted an interaction between several MMPs and integrin β1.

Metastatic breast cancer remains one of the most devastating cancers, and very few new treatments have revealed meaningful improvements in the survival of advanced stage breast cancer patients. Elevated MMP expression and activity within the degradome is characteristic of metastatic breast tumors and is associated with the development of distant metastases. Even though preclinical studies examining the effectiveness of MMP inhibition were encouraging, clinical studies turned out to be disappointing. We showed for the first time that inhibition of MMPs reduced the cell–ECM adhesion molecule integrin β1 expression. We also showed that re-expression of the cell–cell adhesion molecule E-cadherin reduced the active form of integrin β1 along with cell migration. We suggest that a treatment strategy that targets critical nodes in the adhesion-degradome network might be the most effective for clinical translation. Elevated MMP levels may be used to identify and monitor women at high risk of developing metastatic disease.

A quarter of the collagen residues in the tumor microenvironment are proline residues.^11^ Therefore, collagen degradation releases a significant amount of proline, which is in turn used in cellular metabolism as a source of energy. Proline can be metabolized by proline oxidase to generate reactive oxygen species as signaling molecules for epigenetic reprogramming, which regulates the redox homeostasis of cancer cells.^11,49^ It was recently shown that metabolic flux can cause changes in cancer cell adhesion and metastatic transformation.^50^ Future studies should focus on the relationship of the metabolome with the adhesome-degradome network in terms of driving breast cancer metastases.

In summary, our study shows for the first time that cancer cell adhesome and degradome interact in metastatic breast cancer cells, and are modulated during migration and invasion of these cancer cells. These results suggest that targeting nodes in the adhesome-degradome network of breast cancer may be an effective strategy for treating metastatic breast cancer.
