# Supplementary material for: Breast cancer cell adhesome and degradome interact to drive metastasis
Source: NPJ Breast Cancer. 2015 Oct 28;1:15017–. doi: 10.1038/npjbcancer.2015.17 (PMC5515192; doi:10.1038/npjbcancer.2015.17)
Supplement: Supplementary Information [file npjbcancer201517-s1.pdf]

Supplementary Methods

Microarray datasets and bioinformatics analysis

We have used microarray datasets from the Gene Expression Omnibus (GEO) of the National Center for Biotechnology Information (NCBI) of the U.S. National Library of Medicine (http://www.ncbi.nlm.nih.gov/geo/) to search publicly available datasets from human breast cancer cell lines using up-to-date whole-genome microarray chips.

We used the GSE16795 dataset for comparing gene expression levels between metastatic and non-metastatic human breast cancer cell lines [1] . From this dataset, we analyzed 17 metastatic breast cancer cell lines, which were BT-20, BT-549, DU-4475, HCC-1937, Hs578-T, MDA-MB-157, MDA-MB-275VII, MDA-MB-231, MDA-MB-361, MDA-MB-435s, MDA-MB-436, MDA-MB-468, SK-BR-7, SUM102, SUM149, SUM159, and SUM1315. For comparison, we analyzed 11 non-metastatic breast cancer cell lines from this dataset, which were BT-474, T-47D, MCF-7, ZR-75-1, SUM44, SUM52, MPE600, MDA-MB-415, MDA-MB-134VI, CAMA-1, and BT-483. The cells were grown to optimal densities for RNA extraction and hybridization on Affymetrix Human Genome U133A microarrays [1] .

We used the GSE2603 dataset for comparing gene expression levels between wild-type metastatic MDA-MB-231 breast cancer cells (n=2, id: 49953, 49954) and a subpopulation of MDA-MB-231 breast cancer cells (n=4, id: 4142, 4173, 4175, 4180) that exhibited increased lung metastatic tropism when injected into immunodeficient mice [2].

The heat map representations of gene expression were generated using the publicly available software GENE-E from http://www.broadinstitute.org/cancer/software/GENE-E/. The Kaplan–Meier curves of relapse-free survival times of human breast cancer patients were plotted using the KM Plotter software from http://kmplot.com [3]. Kaplan Meier plotter database is capable of assessing the effects of 22,277 genes on survival using 10,188 cancer samples [3, 4]. The protein interaction network was generated using the STRING database version 9.1 [5]. Visualization in the included functional associations and physical interactions were filtered with a setting of >0.70.

Cell lines

We have generated constitutively tdTomato-expressing breast cancer cell lines as listed in Supplementary Figure 1A by using a lentiviral expression vector and fluorescence-activated cell sorting (FACS) as shown in Supplementary Figure 1B-1C.

Briefly, lentiviral vector expressing the tdTomato fluorescent protein under the control of the pGK promoter along with the ΔR8.2 packaging vector and the vesicular stomatitis virus (VSV-G) envelope vector were used to transfect 293T cells. Forty-eight hours post-transfection, virions present in the cell culture media were collected and concentrated. Concentrated virus was used to transduce MCF-7, T-47D, MDA-MB-231, and SUM159 breast cancer cells in the presence of 8μg/ml of polybrene (Sigma, St. Louis, MO). This process was repeated three times to obtain maximum transduction efficiency.

Further, to generate a pool with high intensity fluorescence, transduced cells were sorted by fluorescence-activated cell sorting (FACS) as described in our previous study [6]. Briefly, three weeks following the expansion of tdTomato-fluorescent cells, FACS was performed at The Johns Hopkins University Flow Cytometry Core Facility using a FACS Vantage SE (Becton Dickinson, Franklin Lakes, NJ) instrument. Fluorescence gating was done on the brightest cells, which were collected and then expanded. Cancer cells were cultured in standard medium according to the recommendations of the American Type Culture Collection (ATCC) as summarized in Supplementary Table 2.

The circulating tumor cells from blood and lung metastatic nodules were cultured in cell culture dishes for 2 weeks. The cancer cells were then FACS sorted based on their tdTomato expression.

The tdTomato fluorescent protein falls into the “water window” of low tissue absorption and has a superior quantum yield of 0.69, which is the highest among the available fluorescent proteins in that wavelength range [6]. The tdTomato-fluorescence can be used to monitor tumor growth and metastasis formation, and to isolate and sort circulating tumor cells obtained from tumor-bearing mice.

WST-1 assay

Cell viability was determined by cell proliferation reagent WST-1 (Roche# 11644807001) assay in a 96-well plate format. The wells were treated with 100 µl of collagen 1 (50 µg/ml, Rat Tail, Gibco, A1048301), or 100 µl of matrigel (200ug/ml, Geltrex™ LDEV-Free Reduced Growth Factor Basement Membrane Matrix, Gibco, A1413201) or 100 µl of PBS for 1 hour at 37°C. The plates were then washed twice with PBS. Cells were plated at a density of 5000 cells per well in 100 µl RPMI1640 medium, and cultured for 1-4 days. A separate plate, with three repeats for each sample, was used each day. 10 µl of WST-1 cell proliferation reagent (Roche, Basel, Switzerland) was added, samples were incubated at 37°C for 2 hours, and absorbance was measured at 490 nm. The resulting data are presented after statistical processing.

Clonogenic assays

Cells were trypsinized to produce a single-cell suspension. Cells were then seeded in 6-well culture plates at 100 cells per well. Cells were maintained in culture for 2 weeks, with medium change every 3 days, to let the viable cells propagate to sizable colonies for quantification. The colonies were fixed with 4% paraformaldehyde (4%PFA) and then stained with 0.5% crystal violet for 30 minutes at room temperature. The number of colonies formed in each well was counted and photographed under the microscope. The experiment was repeated three times, and the resulting data are presented after statistical processing.

Protein extraction and Western blotting

Cell pellets were lysed with lysis buffer consisting of 25 mM Tris-HCl pH 7.6, 150 mM NaCl, 1% NP-40, 1% sodium deoxycholate, 0.1% SDS (Thermo Scientific), and protease inhibitor cocktail (1:100, Sigma-Aldrich P8340). Protein concentrations were determined using the bicinchoninic acid assay (BCA Protein Assay Kit, Pierce). A total of 10-20 microgram of proteins were separated by electrophoresis using a NuPAGE 4-12% Bis-Tris Gradient Gel (Invitrogen) and transferred to an Immun-Blot PVDF Membrane (BioRad). Membranes were blocked in 5% milk in Tris-buffered saline - Tween 20 buffer. Membranes were then immunoblotted with anti-MMP-8 antibody (Abcam, ab53017) at a 1:1,000 dilution, anti-MMP-9 antibody (Abcam ab38898) at a 1:1,000 dilution, anti-integrin β1 antibody (Abcam ab52971) at a 1:500 dilution, anti-E-cadherin antibody (Abcam ab1416) at a 1:1,000 dilution, and anti-β-actin antibody (Sigma A1978) at a 1:5,000 dilution. Bound primary antibodies were visualized with appropriate horseradish peroxidase-conjugated secondary antibodies (GE HealthCare Life Science, Mouse-NA931V, Rabbit-NA934V) using enhanced chemiluminescence reagent (Thermo Scientific).

Cell migration and invasion using transwell (Boyden chamber) assays

In the transwell cell migration and invasion assays [7], $1x10^5$ cells were allowed to migrate without any coating and invade with matrigel coating across the transwell inserts, which were cell culture inserts for 24-well plates made from transparent polyethylene terephthalate (PET) with membranes containing pores of 8 µm pore size (BD science). 10% serum was used in the lower chamber as a chemoattractant. For the invasion assays, matrigel was diluted using serum free cold RPMI1640 at a concentration of 200 µg/ml. 100 µl of the diluted matrigel was plated into the transwell and was incubated for 60 minutes at 37°C for gelling. The total growth area in the transwell is about 0.33 cm^2. The experiments were run over 48 hours at 37°C, and repeated three times. Cells from the upper side of the membrane that had not migrated were removed by washing. The migrated cells in the lower surface of the membrane were fixed and stained. The numbers of cells in 5 randomly chosen 1 mm^2 fields of view per experiment were counted to quantify the extent of migration and invasion. Statistical analysis of the data was performed using the paired Student's t-test.

Analysis of MMP-2 and MMP-9 activities by gelatin zymography

To profile secreted gelatinases, gelatin gel zymography was performed as per the manufacturer's instructions (Life Technologies). In brief, when the cells reached around 80% to 90% confluence, the medium was changed to serum free medium. Conditioned medium was collected after 24 hours. Equal 10 µl volumes of conditioned medium were mixed with Tris-Glycine SDS Sample Buffer (2X) (1:1, v/v) and were electrophoresed under non-reducing conditions in 10% Tris-Glycine gel with 0.1% gelatin as the substrate. PageRuler Plus Prestained Protein Ladder (Thermo Scientific #26616) was used as size standard. After electrophoresis, the gel was incubated with zymogram renaturing buffer (Life Technologies, LC2670) for 30 minutes at room temperature with gentle agitation. Then, the zymogram renaturing buffer was discarded and zymogram developing buffer (Life Technologies, LC2671) was added and equilibrated for 30 minutes at room temperature with gentle agitation. After that, fresh zymogram developing buffer was added and the gel was incubated overnight at 37°C. The gels were stained with Coomassie blue stain (Life Technologies LC6060).

Adhesion assay

300µl of collagen 1 at a concentration of 50 µg/ml or matrigel at a concentration of 200µg/ml or PBS were added into the wells of 24-well plates and were incubated at 37°C for 1 hour. The wells were then washed with PBS. The cancer cells were deprived of serum for 12 hours before

the adhesion assay. Cells were detached using cell dissociation solution TrypLE™ Express (Life technology 12604). After detachment, the cells were washed twice with serum free medium. 500 µl of cell suspension containing 500,000 cells in serum free medium were added to each well and incubated for 30 minutes at 37°C. Non-adherent cells were removed by washing with serum-free medium. The remaining attached cells were fixed with 4% PFA and stained with 0.5% crystal violet for 30 minutes at room temperature. All assays were conducted in triplicate.

Quantification of adherent cells

96-well plates were used for the quantification of the number of adherent cells. 100 µl of collagen 1 at a concentration of 50 µg/ml or matrigel at a concentration of 200µg/ml or PBS were added into the wells of 96-well plates and were incubated at 37°C for 1 hour. The wells were then washed with PBS. The cancer cells were deprived of serum for 12 hours before the adhesion assay. The cells were detached using cell dissociation solution TrypLE™ Express (Life technology 12604). After detachment, cells were washed twice with serum free medium. 100 µl of cell suspension containing 100,000 cells in serum free medium were added to each well and incubated for 30 minutes at 37°C. Non-adherent cells were removed by washing with serum-free medium. After washing off the non-adherent cells, 100 µl of cell culture medium with 10% serum were added and cells were incubated at 37 °C for 4 h for recovery. Then, 10 µl of WST-1 substrate was added to each well and incubated for an additional 2 h at 30 °C. The numbers of viable cells were measured at 490 nm (absorbance). The resulting data are presented after statistical processing.

Hanging drop assay

This assay is used for assessing aggregation properties of cancer cells. 15 µl drops of cell suspensions containing 250,000 cells per ml were pipetted onto the inner surface of the lid of a 12-well culture plate. The lid was quickly flipped over and then placed on the plate so that the drops were hanging from the lid with the cells suspended within them. To prevent evaporation, 2 ml of serum-free culture medium was placed in the well. After 24 hours at 37 °C, the lid of the plate was inverted and photographed using a ZEISS inverted tissue culture microscope at 20x magnification. At least 5 drops were analyzed per experiment.

Hanging drop assay with fibroblasts

Human mammary fibroblasts were a kind gift from Dr. Gary Luker at the University of Michigan [8]. HMF cells were stained with the Qtracker® 525 Cell Labeling Kit as per the manufacturer's

protocol (Life Technologies, Q25041MP). Cancer cells and mammary fibroblast cells were mixed at a 1:1 ratio and the hanging drop assay were performed as described above. After 24 hours at 37 °C, the tdTomato-expressing cancer cells and the green fluorescent dye labeled fibroblast were imaged using the ZEISS inverted microscope using the filter wheel at the TRITC and FITC position, respectively.

Cell Aggregation assay in collagen 1 gel

24-well plate wells were coated with a layer of neutralized collagen 1 gel as per the manufacturer's instructions using rat tail collagen 1 (Gibco, A1048301), dH_20, PBS, and NaOH. In brief, 0.5 ml of collagen 1 gel were added at a concentration of 1 mg/ml to each well of a 24-well plate and incubated for 2 hours at 37 °C to allow fibril formation. After washing with water and drying of the collagen gels at room temperature, 50,000 cells in 30 µl medium were seeded onto each gel. Five hours later, standard cell culture medium was added. After incubation for 3 days at 37°C, cell aggregation was imaged with an inverted bright field microscope.

E-Cadherin transfection

pcDNA3.1-E-cadherin-GFP was a gift from Jennifer Stow (Addgene plasmid # 28009) [9]. pcDNA3-EGFP was a gift from Doug Golenbock (Addgene plasmid # 13031). For transfection and expression of cDNAs, $5X10^5$ cells each were placed in 6-well plates for 24 hours prior to use, and 4 µg of plasmid encoding human E-cadherin-GFP or EGFP were added. Lipofectamine 2000 Transfection Reagent (Life Technology# 11668027) was added according to the manufacturer's protocol (http://www.lifetechnologies.com/order/catalog/product/11668027). For stably expressing lines, transfected cells were passaged and maintained in media containing G418 (Geneticin, Life Technologies# 10131035). Cells were kept under selection for 7-10 days. Then, the cells from the selection step were plated at a density of 10 cells per ml in a 96-well tissue culture plate adding 100 µl per well (i.e., 1 cell per well). Selected single-colony wells in the 96-well tissue culture plate were expanded to high confluence and transferred to a 24-well tissue culture plate. Once the colonies in 24-well tissue culture plates were expanded to high confluence, they were passaged to a 6-well tissue culture plate. Clonal cell lines were assessed by qRT-PCR to select lines with increased levels of E-cadherin gene expression.

qRT-PCR

For RNA purification, cells were grown for 48 hours during the exponential growth phase. RNA was isolated using the RNeasy total RNA isolation kit (Qiagen, Germantown, MD) according to

the manufacturer's protocol and were reverse transcribed to cDNA using qScript™ cDNA SuperMix (Quanta Bioscience, Gaithersburg, MD). Quantitative real-time PCR (q-RT-PCR) was performed using IQ SYBR Green Supermix and gene-specific primers in the iCycler RT-PCR detection system (Bio-Rad, Hercules, CA) with 2 µl of diluted cDNA samples at a 1:10 dilution used as a template using the following primer: hEcad_qrtpcr Primer Forward: CAGAAAGTTTTCCACCAAAG; Reverse: AAATGTGAGCAATTCTGCTT [10]. The expression of CDH1 mRNA relative to the housekeeping gene hypoxanthine phosphoribosyltransferase 1 (HPRT1) was calculated based on the threshold cycle (Ct) as $R = 2^{-\Delta(\Delta Ct)}$, where $\Delta Ct = Ct_{target} - Ct_{HPRT1}$ and $\Delta(\Delta Ct) = \Delta Ct_{ECadherin\text{-}GFP} - \Delta Ct_{EGFP}$ [11].

Breast tumor xenograft models

All animal experiments were approved by the Institutional Animal Care and Use Committee. 2×10^6 breast cancer cells were orthotopically implanted into the fourth right mammary fat pad of 6 weeks old female athymic nu/nu mice (NCI) as described previously [7]. Mice with BT-474, T-47D, and MCF-7 tumor xenografts were implanted with a 60-day release pellet containing 0.72 mg 17β-Estradiol per pellet (Innovative Research of America, SE#121) in the neck region at 24 hours prior to tumor inoculation [12]. Primary tumor volumes were calculated from caliper measurements of tumors using the formula Volume = $(4/3) \times 3.14 \times (\text{radius})^3 = (3.14/6) \times (a.b.c)$, where a, b, and c are three measured orthogonal diameters of the tumor [7]. Additionally, primary tumor growth and metastasis formation were optically tracked once or twice a week by tdTomato fluorescence imaging using a Xenogen IVIS 200 optical imaging system. All mice were sacrificed at 8 to 12 weeks following orthotopic inoculation when primary tumors reached sizes of 500-1000 mm^3. 5 mice were used per group.

Necropsy

Each mouse was imaged at 24 hours after the injection of MMPSence and AngioSense. Then, animals were sacrificed and tumor xenografts and lungs were excised for *ex vivo* fluorescence imaging. Four to six 2-mm thick fresh tissue sections were cut from the primary tumor using an adjustable tissue slicer (Braintree Scientific). Tumor sections and whole lungs were imaged with IVIS camera settings as described in the 'Methods' section. After the imaging experiments, all tumor sections and lungs were washed in PBS, fixed in 10% formalin for 24 hours, and embedded in paraffin blocks.

Immunohistochemistry

All tissues were paraffin-embedded, serially sectioned into 5 to 20 sections at 5 μm thickness, and mounted onto microscopy slides by the 'Histology Laboratory' Facility of the Molecular & Comparative Pathobiology Department of the Johns Hopkins Medical Institutions. Tissues were serially sectioned from the mid-section of the respective tissue at its maximum diameter. Hematoxylin and eosin (H&E) staining was performed by the 'Histology Laboratory' Facility as well. Unstained sectioned were further processed for immunohistochemical detection of MMP-1, MMP-9, and ITGB-1.

All sections for immunohistochemistry were deparaffinized and hydrated using graded concentrations of ethanol to deionized water. Anti-MMP-1 antibody (Abcam ab137332) at a 1:100 dilution, anti-MMP-9 antibody (Abcam ab38898) at a 1:100 dilution, and anti-integrin-β1 antibody (Abcam ab52971) at a 1:200 was used as the primary antibody for overnight incubation at 4°C. Signals were visualized using the Signal Stain Boost IHC Detection Reagent (Cell signaling- Rabbit#8814, Mouse#8125 Rabbit), and DAB Quanto Chromogen and Substrate (Thermo Scientific, TA-060-QHDX). Finally, slides were counterstained with hematoxylin, and dehydrated through a series of washes in xylene and ethanol. The slides were mounted with coverslips using Permount (catalog number SP15, Fisher Scientific, Waltham, MA). Control slides were stained keeping all the steps the same without adding primary antibody or Signal Boost Detection Reagent [13].

References

1. Hollestelle, A. *et al.* Distinct gene mutation profiles among luminal-type and basal-type breast cancer cell lines. *Breast Cancer Res Treat* **121**, 53-64 (2009).
2. Minn, A.J. *et al.* Genes that mediate breast cancer metastasis to lung. *Nature* **436**, 518-524 (2005).
3. Gyorffy, B. *et al.* An online survival analysis tool to rapidly assess the effect of 22,277 genes on breast cancer prognosis using microarray data of 1,809 patients. *Breast Cancer Res Treat* **123**, 725-731 (2009).
4. Gyorffy, B., Lanczky, A. & Szallasi, Z. Implementing an online tool for genome-wide validation of survival-associated biomarkers in ovarian-cancer using microarray data from 1287 patients. *Endocr Relat Cancer* **19**, 197-208 (2012).
5. Szklarczyk, D. *et al.* The STRING database in 2011: functional interaction networks of proteins, globally integrated and scored. *Nucleic Acids Res* **39**, D561-568 (2011).
6. Winnard, P.T., Jr., Kluth, J.B. & Raman, V. Noninvasive optical tracking of red fluorescent protein-expressing cancer cells in a model of metastatic breast cancer. *Neoplasia* **8**, 796-806 (2006).
7. Rizwan, A. *et al.* Relationships between LDH-A, Lactate and Metastases in 4T1 Breast Tumors. *Clin Cancer Res* (2013).

8. Orimo, A. *et al.* Stromal fibroblasts present in invasive human breast carcinomas promote tumor growth and angiogenesis through elevated SDF-1/CXCL12 secretion. *Cell* **121**, 335-348 (2005).
9. Miranda, K.C. *et al.* A dileucine motif targets E-cadherin to the basolateral cell surface in Madin-Darby canine kidney and LLC-PK1 epithelial cells. *J Biol Chem* **276**, 22565-22572 (2001).
10. Privette Vinnedge, L.M. *et al.* The human DEK oncogene stimulates beta-catenin signaling, invasion and mammosphere formation in breast cancer. *Oncogene* **30**, 2741-2752 (2011).
11. Krishnamachary, B. *et al.* Hypoxia regulates CD44 and its variant isoforms through HIF-1alpha in triple negative breast cancer. *PLoS One* **7**, e44078 (2012).
12. Yarden, R.I., Lauber, A.H., El-Ashry, D. & Chrysogelos, S.A. Bimodal regulation of epidermal growth factor receptor by estrogen in breast cancer cells. *Endocrinology* **137**, 2739-2747 (1996).
13. Burry, R.W. Controls for immunocytochemistry: an update. *J Histochem Cytochem* **59**, 6-12 (2010).
