# Supplementary material for: Breast cancer cell adhesome and degradome interact to drive metastasis
Source: NPJ Breast Cancer. 2015 Oct 28;1:15017–. doi: 10.1038/npjbcancer.2015.17 (PMC5515192; doi:10.1038/npjbcancer.2015.17)

# Supplementary Figure 1

## A

| Panel of metastatic and non-metastatic breast cancer cell lines and xenografts. Metastatic lines are shown in red and nonmetastatic lines in blue. | | | | | | | |
|---|---|---|---|---|---|---|---|
| Name | Type | ER | PR | ERBB2 | Source | Disease | Metastases (Xenograft) |
| MDA-MB-231 | Basal | - | - | - | Metastatic site | adenocarcinoma | Yes |
| SUM149PT | Basal | - | - | - | Primary breast tumor | inflammatory ductal carcinoma | Yes |
| SUM159PT | Basal | - | - | - | Primary breast tumor | anaplastic carcinoma | Yes |
| BT-474 | Luminal-B | - | + | ++ | Primary breast tumor | ductal carcinoma | No |
| T47D | Luminal-A | + | + | - | Metastatic site | ductal carcinoma | No |
| MCF-7 | Luminal-A | + | + | - | Metastatic site | adenocarcinoma | No |

## B

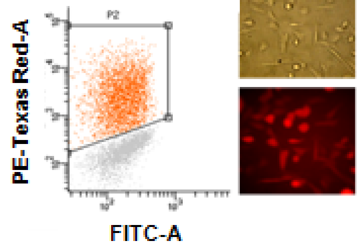


## C

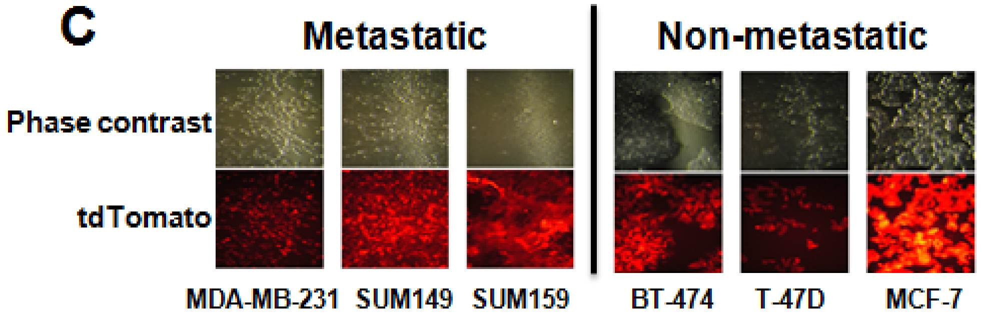


## D

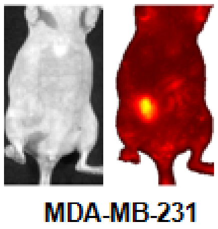


## E

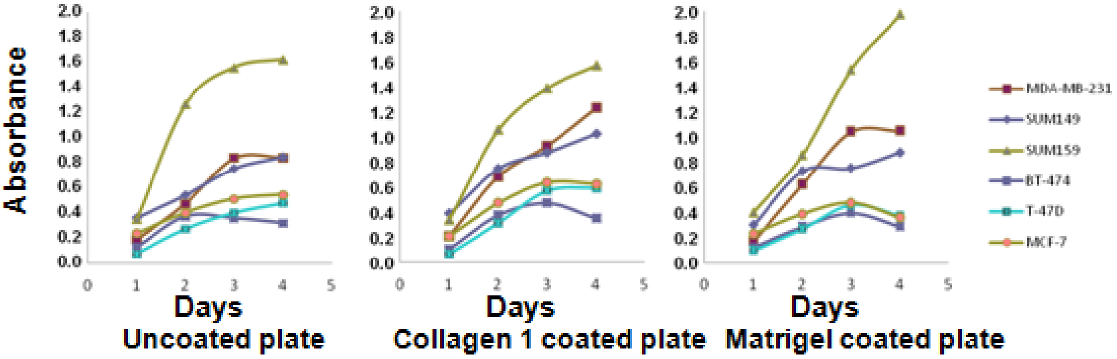

Supplement: Supplementary Figure 1 [file npjbcancer201517-s2.pdf]
