# Supplementary figures and images for: Breast cancer cell adhesome and degradome interact to drive metastasis
Source: NPJ Breast Cancer. 2015 Oct 28;1:15017–. doi: 10.1038/npjbcancer.2015.17 (PMC5515192; doi:10.1038/npjbcancer.2015.17)

## Supplementary Figure 2

**A**

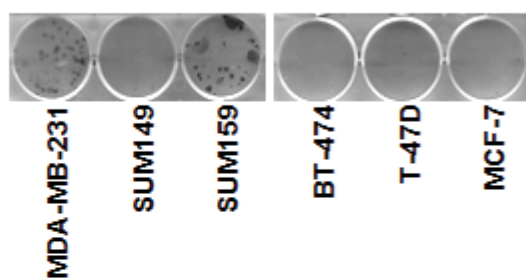

**B**

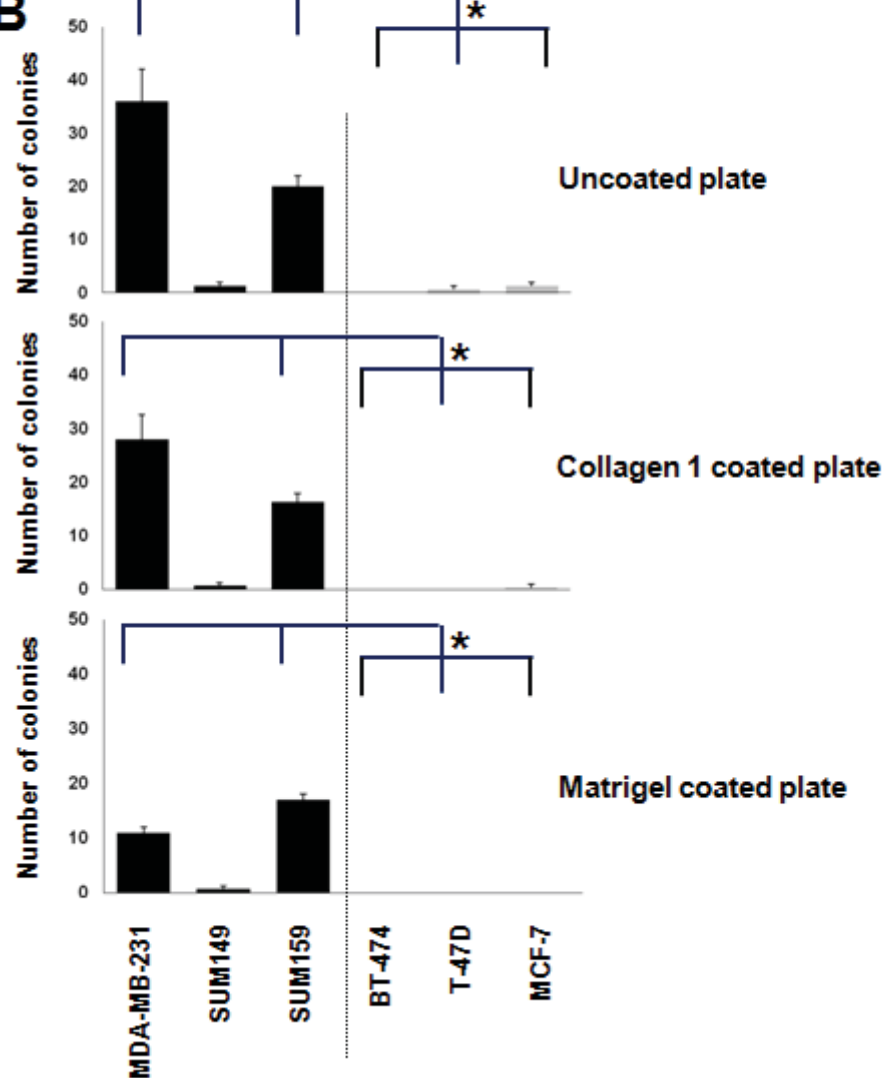

Supplement: Supplementary Figure 2 [file npjbcancer201517-s3.pdf]

## Supplementary Figure 3

**A**

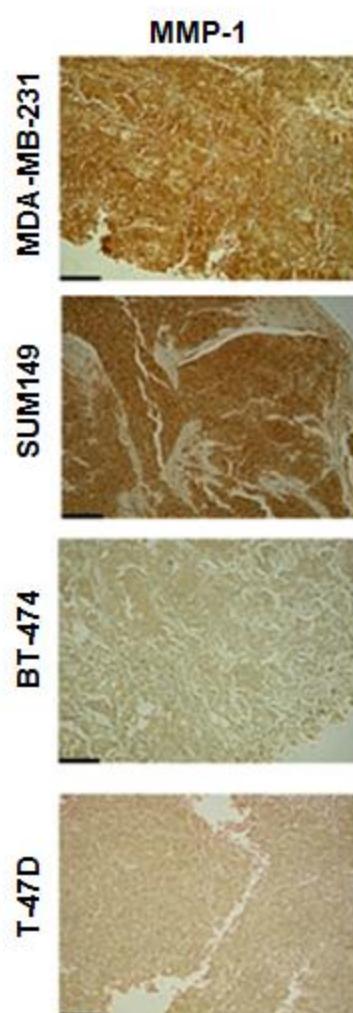

**B**

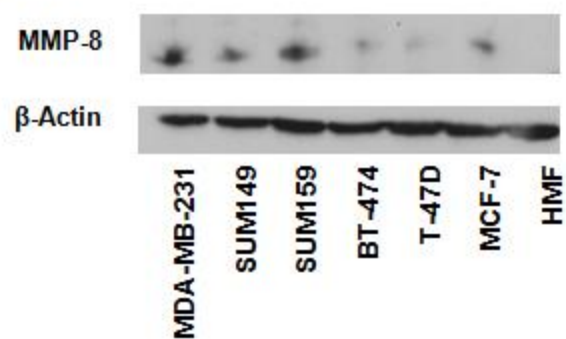

Supplement: Supplementary Figure 3 [file npjbcancer201517-s4.pdf]
