# Supplementary material for: Breast cancer cell adhesome and degradome interact to drive metastasis
Source: NPJ Breast Cancer. 2015 Oct 28;1:15017–. doi: 10.1038/npjbcancer.2015.17 (PMC5515192; doi:10.1038/npjbcancer.2015.17)
Supplement: Supplementary Figure 4 [file npjbcancer201517-s5.pdf]

# Supplementary Figure 4

## Relative CDH1 expression

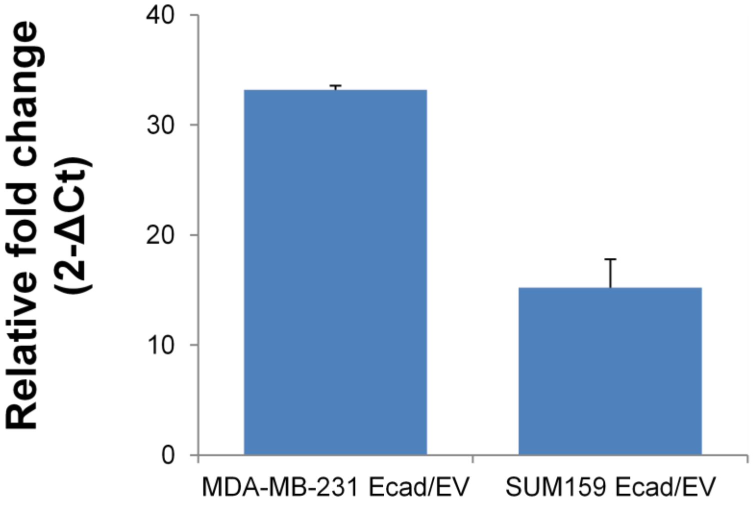


**Primer**
Forward: CAGAAAGTTTTCCACCAAAG
Reverse: AAATGTGAGCAATTCTGCTT

**Ecad: E-cadherin Re-expressed Cell**
**EV: Empty Vector**
