# Supplementary material for: Breast cancer cell adhesome and degradome interact to drive metastasis
Source: NPJ Breast Cancer. 2015 Oct 28;1:15017–. doi: 10.1038/npjbcancer.2015.17 (PMC5515192; doi:10.1038/npjbcancer.2015.17)
Supplement: Supplementary Table 1 [file npjbcancer201517-s6.pdf]

Supplementary Table 1

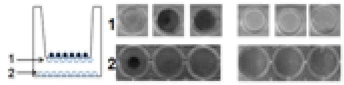

Migration-1 (Attach to bottom of membrane)

	MDA-MB-231	SUM149	SUM159	BT-474	T-47D	MCF-7
MDA-MB-231		*	*	*	*	*
SUM149			*	*	*	*
SUM159				*	*	*
BT-474					NS	NS
T-47D						NS
MCF-7						

Migration-2 (Falls to bottom of the well)

	MDA-MB-231	SUM149	SUM159	BT-474	T-47D	MCF-7
MDA-MB-231		*	*	*	*	*
SUM149			*	*	*	*
SUM159				*	*	*
BT-474					NS	NS
T-47D						NS
MCF-7						

Invasion-1 (Attach to bottom of membrane)

	MDA-MB-231	SUM149	SUM159	BT-474	T-47D	MCF-7
MDA-MB-231		*	*	NS	NS	NS
SUM149			*	*	*	*
SUM159				*	*	*
BT-474					NS	NS
T-47D						NS
MCF-7						

Invasion-2 (Falls to bottom of the well)

	MDA-MB-231	SUM149	SUM159	BT-474	T-47D	MCF-7
MDA-MB-231		*	*	*	*	*
SUM149			*	*	*	*
SUM159				*	*	*
BT-474					NS	NS
T-47D						NS
MCF-7						

NS= Not significant, *= Significant
