# Supplementary material for: Breast cancer cell adhesome and degradome interact to drive metastasis
Source: NPJ Breast Cancer. 2015 Oct 28;1:15017–. doi: 10.1038/npjbcancer.2015.17 (PMC5515192; doi:10.1038/npjbcancer.2015.17)
Supplement: Supplementary Table 2 [file npjbcancer201517-s7.pdf]

Supplementary Table 2

Cell Line	Basal Medium	Additives*
MDA-MB-231	RPMI 1640 (Sigma-Aldrich)	10% FBS
SUM149	RPMI 1640 (Sigma-Aldrich)	10% FBS/Ins/Hyd
SUM159	RPMI 1640 (Sigma-Aldrich)	10% FBS/Ins/Hyd
BT-474	RPMI 1640 (Sigma-Aldrich)	10% FBS/E2
T-47D	RPMI 1640 (Sigma-Aldrich)	10% FBS/E2
MCF-7	RPMI 1640 (Sigma-Aldrich)	10% FBS/Ins

Ins = Insulin; Hyd = Hydrocortisone, E2=beta-Estradiol
