# Supplementary material for: Breast cancer cell adhesome and degradome interact to drive metastasis
Source: NPJ Breast Cancer. 2015 Oct 28;1:15017–. doi: 10.1038/npjbcancer.2015.17 (PMC5515192; doi:10.1038/npjbcancer.2015.17)
Supplement: Supplementary Legends [file npjbcancer201517-s8.doc]

**Legends of Supplementary Figures**

**Supplementary Figure 1:(A)**Panel of metastatic and non-metastatic breast cancer cell lines and xenografts. Metastatic lines are shown in red and non-metastatic lines are shown in blue.**(B)**Fluorescence gating used in FACS-sorting of tdTomato-expressing MDA-MB-231 cells (red).**(C)**Photographs and fluorescence images of breast cancer cell lines expressing tdTomato*in vitro*. **(D)**Representative photographs of a mouse with an orthotopictdTomato-expressingMDA-MB-231 breast tumor *in vivo*. (E) WST-1 cell proliferation assay with tdTomato-expressing breast cancer cell lines in complete medium with 10% FBS in uncoated, collagen1 coated, and matrigel coated plates.

**Supplementary Figure 2:(A)**Photographs of a representative clonogenic assays from a 6 well plate after 2 weeks at a seeding density of 100 cells per well, which was performed with tdTomato-expressing breast cancer cell lines.**(B)**Quantification of clonogenic assays. The plates were uncoated, collagen1 coated, or matrigel coated.

**Supplementary Figure 3:(A)** Immunohistochemistry (IHC) analysis demonstrated strong MMP-1 expression in metastatic MDA-MB-231 and SUM149 tumor sections, but not in non-metastatic BT-474 and T-47D tumor sections. Scale bar, 200 μm. **(B)** Western blots showing MMP-8 expression in breast cancer cell lines. High MMP-9 expression was observed in metastatic lines, while non-metastatic lines and fibroblasts expressed small amounts or no MMP-8. ß-actin was used as loading control.

**Supplementary Figure 4:**Relative fold change (2-ΔCt) in E-cadherin (CDH1)mRNA expression in E-cadherin re-expressing MDA-MB-231 and SUM159 cells when compared with cells transfected with empty vectors. Two sets of primer were used. Primer #1:Forward:CAGAAAGTTTTCCACCAAAG,Reverse:AAATGTGAGCAATTCTGCTT. Primer # 2: Forward: GAAGGTGACAGAGCCTCTGGAT, Reverse: GATCGGTTACCGTGATCAAAATC.

**Supplementary Table1:**A one-way analysisof variance (ANOVA) was calculated from the number of cells that have migrated or invaded in all experimental groups. Post-hoc comparisons using the Fisher Least Significant Difference (LSD) were explored to compare the mean of one group with the mean of another group. p< 0.05 was considered statistically significant and is indicated in all figures with a *.

**Supplementary Table 2:**Cell type specific media used for breast cancer cell lines. All media contain 100 U/ml penicillin, 100 µg/ml streptomycin. Ins = Insulin; Hyd = Hydrocortisone, E2=beta-Estradiol
